# Diclofenac-Impregnated Mesoporous Carbon-Based Electrode Material for the Analysis of the Arsenic Drug Roxarsone

**DOI:** 10.3390/ma16155420

**Published:** 2023-08-02

**Authors:** Katarzyna Tyszczuk-Rotko, Damian Gorylewski, Rafał Olchowski, Ryszard Dobrowolski

**Affiliations:** 1 Faculty of Chemistry, Institute of Chemical Sciences, Maria Curie-Skłodowska University in Lublin, 20-031 Lublin, Poland; 2 Department of Pharmacology, Toxicology and Environmental Protection, Faculty of Veterinary Medicine, University of Life Sciences, 20-950 Lublin, Poland

**Keywords:** mesoporous material, stripping voltammetry, roxarsone, river water, wastewater

## Abstract

This paper describes a novel electrode material, diclofenac-impregnated mesoporous carbon modified with a cationic surfactant, cetyltrimethylammonium bromide (DF-CMK-3/CTAB), for ultratrace analysis of the arsenic drug roxarsone (ROX). DF-CMK-3 amorphous carbon is a material with a high specific surface area and well-defined, hexagonally ordered, thin mesopores. The functional groups attached to the carbonaceous surface, such as chromene and pyron-like oxygen groups, lactam, and aromatic carbon rings, have the basic character and they can donate electrons. Modification of DF-CMK-3 with a CTAB layer significantly increases the analytical signal due to electrostatic interactions between the cationic surfactant and the anion form of ROX in the acidic medium. The voltammetric procedure at the glassy carbon sensor modified with DF-CMK-3/CTAB exhibited excellent sensitivity (limit of detection of 9.6 × 10^−11^ M) with a wide range of linearity from 5.0 × 10^−10^ to 1.0 × 10^−4^ M. Analysis of real samples (treated municipal wastewater and river water) showed recoveries from 96 to 102% without applying the complicated sample pretreatment step. The sensor demonstrated excellent sensitivity in the analysis of the arsenic drug ROX in the presence of interferences in environmental water samples.

## 1. Introduction

3-nitro-4-hydroxyphenyl arsenic acid, known as roxarsone (ROX), has been used for many years in veterinary medicine as a drug against coccidiosis—a parasitic disease of poultry—and dysentery. It has also been used as a feed additive that supports the growth of poultry and swine [1,2,3]. The greater part of ROX consumed by poultry has been shown to be excreted in unchanged form, but small amounts of this drug accumulate in animal bodies. In animal excrements, arsenic contained in roxarsone is converted into toxic inorganic compounds of As(III) and As(V). Therefore, it contributes to the contamination of the natural environment and may pose a direct threat to humans. For this reason, the use of this compound in the production of feed for poultry and swine was banned by the EU (1999) and China (2019), and otherwise discontinued in the USA (2011) [4,5,6]. Unfortunately, the use of ROX for the production of animal feed has not been suspended in many parts of the world, i.e., certain parts of Asia and South America, as well as Canada [5,7]. It is worth mentioning that a lot of foodstuffs are imported from abroad. Hence, the potential risk of coming into contact with roxarsone is also real for people who do not live in the regions where ROX is still in use. For this reason, it is important to develop sensitive methods for detecting this compound at trace levels.

Many methods for the determination of ROX have been described over the years. Many of them are chromatographic methods such as high-performance liquid chromatography (HPLC) and gas chromatography (GC) [8,9]. Many voltammetric procedures for ROX determination have been presented in the literature [10,11,12,13,14,15,16,17,18,19,20,21,22,23]. Voltammetric methods are characterized by very high sensitivity. In the adsorptive stripping voltammetry (AdSV) procedure, for example, the additional step of analyte adsorption onto the surface of the working electrode before the registration of the analytical signal allows very low limits of detection to be achieved, and these limits of detection are out of range for most other analytical methods. Moreover, the great advantages of voltammetric methods include low consumption of reagents during measurements, portability, relatively low cost of apparatus compared with, e.g., chromatographic methods, and a short duration time of the analysis. They can be used as a complementary tool for analysis performed using other analytical techniques. Additionally, there is a possibility of performing some voltammetric measurements using on-site analysis [17].

Mesoporous carbonaceous materials such as CMK-3 have a lot of advantages, e.g., a considerable pore volume, a large active surface area, and great opportunities for chemical modification of the carbonaceous surface by the intersection of various kinds of heteroatoms such as nitrogen, phosphorus, sulfur, oxygen, or boron. They also have good thermal resistance and very high mechanical and chemical stability [24,25,26,27,28,29]. It is worth mentioning that CMK-3 materials have good electrical conductivity and they can therefore be successfully used in electrochemical measurements; for example, dicyandiamide-impregnated mesoporous carbonaceous material additionally electrochemically decorated with lead film (Pb-N-CMK-3) was used as a glassy carbon electrode (GCE) modifier in the case of U(VI) determination [30].

In our previous work, a new sensitive sensor for trace analysis of roxarsone was presented: a glassy carbon electrode modified with a cationic surfactant, cetyltrimethylammonium bromide (GCE/CTAB), in combination with square-wave adsorptive stripping voltammetry (SWAdSV) [20]. In this study, we attempted to improve the sensitivity of this sensor by firstly modifying the GCE with diclofenac-impregnated mesoporous carbon material (DF-CMK-3), and secondly, by the deposition of CTAB onto the surface of the new sensor. Diclofenac molecules have some heteroatoms (N, O) that can be built into the structure of CMK-3 carbon during its thermochemical modification. These heteroatoms can enhance the electrical properties of carbon material [25]. The choice of the surfactant was suggested based on the experience from our previous study [20] that investigated the influence of cationic, anionic, and neutral surfactants on the ROX electroreduction signal. The analytical signal was observed to significantly increase only in the case of the presence of the cationic surfactant (CTAB) in the base electrolyte due to electrostatic interactions between the cationic surfactant and the anion form of roxarsone in the solution with acidic pH [31].

In this way, a new sensor (GCE/DF-CMK-3/CTAB) with very good conductive properties and a large active surface was obtained. It was also shown that this sensor, combined with the square-wave adsorptive stripping voltammetric (SWAdSV) technique, achieves satisfactory analytical parameters and allows ROX to be determined in a sample of river water and wastewater at a trace level.

## 2. Materials and Methods

### 2.1. Reagents and Solutions

Synthesis of DF-CMK-3: diclofenac sodium (Sigma-Aldrich, St. Louis, MO, USA) and distilled water (Merck, Darmstadt, Germany) were used during synthesis.

Electrochemical measurements: Appropriate volumes of 1 M CH_3_COONH_4_ and 1 M HCl (Sigma-Aldrich, St. Louis, MO, USA) were used to obtain 1 M buffer solution (CH_3_COONH_4_, CH_3_COOH, and NH_4_Cl) with a pH of 5.6. The standard solutions of Fe^3+^, Cd^2+^, Zn^2+^, Cu^2+^, Ni^2+^, Sb^3+^, Pb^2+^, NO_2_^−^, NO_3_^−^, Cl^−^, and Triton X-100 (Merck, Darmstadt, Germany) were used to study the selectivity of the proposed procedure, and 96% ethanol solution (Merck, Darmstadt, Germany) was used to prepare 10 and 1 mM roxarsone (ROX, AK Scientific, Union City, CA, USA). The ROX solution with a concentration of 0.01 mM was prepared daily in 0.1 M PBS buffer with a pH of 7.2. The wastewater samples were obtained from the Municipal Water Supply & Waste Water Treatment Company Ltd. (Lublin, Poland), while river water samples were collected from the Vistula River (Sandomierz, Poland). The ROX spiked samples were filtered (0.22 µm Millipore filter).

### 2.2. Apparatus

Synthesis and characteristics of DF-CMK-3 and GCE/DF-CMK-3/CTAB: A magnetic stirrer (MS 11 HS, Wigo, Pruszkow, Poland), a laboratory oven (SML 32/250, Zalmer, Warsaw, Poland), and a quartz tubular furnace equipped with a nitrogen cylinder (5N, Air Liquide, Kraków, Poland) were used during the synthesis of the carbon material. The physicochemical characteristics of the synthesized carbonaceous material were analyzed using a nitrogen sorption analyzer (ASAP 2420, Micromeritics Inc., Norcross, GA, USA) with a degassing temperature of 120 °C, a dispersive Raman microscope (inVia Reflex, Renishaw, Wotton-under-Edge, UK) equipped with an argon laser (wavelength: 514 nm, source power: 20 mW), an XRD diffractometer (Empyrean, PANalytical, Malvern, UK) equipped with an X radiation source (CuK_α_), an FT-IR spectrometer (Nicolet 8700A, Thermo Scientific, Waltham, MA, USA) with KBr tabs, a zeta potential analyzer (Zetasizer Nano ZS, Malvern Instruments, Malvern, UK) (2 mg of carbon material with 2 mL of 0.001 mol/L KCl), a pH-meter (N517, Mero Tronik, Warsaw, Poland), a CHN analyzer (EA3000 Elemental WAnalyzer, Euro Vector, Pavia PV, Italy), a SEM microscope (Carl Zeiss Ultra Plus, Carl Zeiss, Jena, Germany) (acceleration voltage: 20 kV, probe current: 5 nA) equipped with an X-ray spectrometer with energy dispersion (EDX) (Bruker AXS, Bruker, Karlsruhe, Germany), and an X-ray photoelectron spectrometer (Prevac, Rogów, Poland) equipped with a monochromatic X-ray source (AlK_α_; 1486.6 eV; Gammadata Scienta, Uppsala, Sweden, source power: 450 W) and calibrated towards C 1s signal at 285 eV.

Electrochemical measurements: Voltammetric (cyclic voltammetry (CV), square-wave voltammetry (SWV)) and electrochemical impedance spectroscopy (EIS) measurements were performed using a conventional three-electrode system consisting of the GCE or DF-CMK-3/GCE (a glassy carbon electrode modified with mesoporous carbon obtained by impregnation with diclofenac and cetyltrimethylammonium bromide, diameter of 1 mm) as working electrodes, Ag/AgCl (3 M KCl) as a reference electrode, and platinum as a counter-electrode, which was coupled with a µAutolab potentiostat/galwanostat (Eco Chemie, Utrecht, The Netherlands) controlled by GPES 4.9 or FRA 4.9 software. The GCE surface was polished daily on silicon carbide paper (SiC-paper, #4000, Buehler, Skovlunde, Denmark), and alumina particle suspensions (0.3 µm) on a Buehler polishing pad, with subsequent washing and sonication for 60 s.

### 2.3. Synthesis of DF-CMK-3

For the synthesis of DF-CMK-3, 1 g of pristine CMK-3 carbon [24] was mixed with 250 mL of 888 mg/L sodium diclofenac aqueous solution. The carbonaceous slurry was mixed (200 rpm) for 7 days at room temperature. Next, the impregnated mesoporous carbon was filtered and dried at 120 °C for 24 h. The obtained solid sample was pyrolyzed in the quartz tubular furnace at 800 °C for 40 min under nitrogen atmosphere. The synthesized carbon was denoted as DF-CMK-3.

### 2.4. GCE/DF-CMK-3/CTAB Preparation and ROX Analysis

Then, 0.4 µL of the DF-CMK-3 suspension (0.5 mg in 100 µL DMF) was applied to the polished and dried GC electrode surface. The modified electrode was left at room temperature for 10 min to evaporate the solvent. Voltammetric ROX determination was carried out in 0.075 M buffer solution (CH_3_COONH_4_, CH_3_COOH, and NH_4_Cl) of pH = 5.6 and 40 mg/L CTAB. Each ROX measurement was carried out after waiting for 90 s, without applying a potential to the working electrode and without mixing the solution. The voltammograms (SWAdSV) were performed in a potential range from −0.1 to −1.1 V with a frequency (f) of 200 Hz, an amplitude (E_SW_) of 75 mV, and a step potential (ΔE) of 6 mV. The background was subtracted from each measurement and background corrections were made.

## 3. Results and Discussion

### 3.1. Physicochemical Characteristics of DF-CMK-3

**Figure 1:**
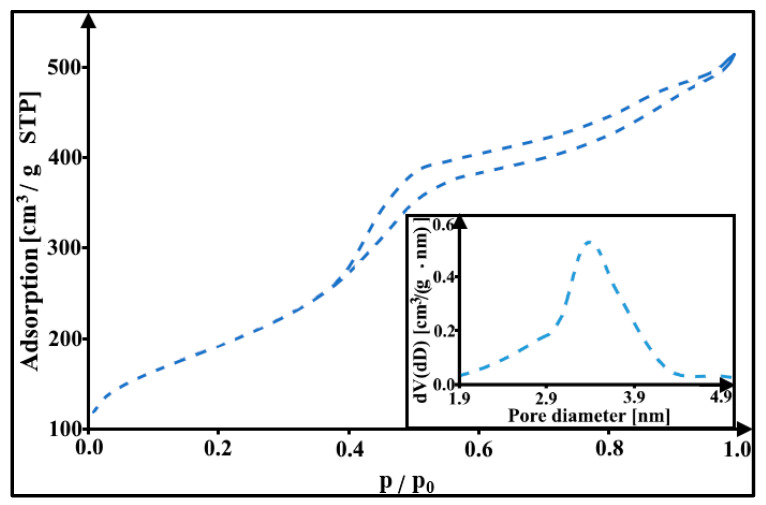
The adsorption/desorption isotherm and pore size distribution (inlet) for the DF-CMK-3 carbon.

Figure 1 shows the nitrogen adsorption/desorption isotherm and the pore size distribution for the DF-CMK-3 carbon. The presented isotherm can be classified as type IVa according to the IUPAC. In this isotherm, the hysteresis loop of type H1 can be seen, which begins from a relative pressure of 0.39 [32]. It indicates the presence of homogenous, narrow, and slit-shaped mesopores in the porous structure of the carbonaceous material studied. The additional confirmation of the occurrence of the mesopores in the DF-CMK-3 structure is the pore size distribution with a maximum of 3.4 nm. The porosity parameters for the studied carbon material were also estimated from the isotherm obtained using the BET and BJH methods and they are given in Table 1. The CMK-3 carbon thermochemically modified with sodium diclofenac is characterized by a high specific surface area (690 m^2^/g), which can be associated with the presence of micropores in its structure. Again, the presence of mesopores in the DF-CMK-3 carbon is evidenced by its high total pore volume (0.79 cm^3^/g) and pore diameter (3.4 nm), located in the range of 2–50 nm.

The zeta potential and pH of 1 mM KCl for the studied carbon immersed in this electrolyte were determined (Table 1) as 12.3 mV and 7.77, respectively, and indicate the basic character of the DF-CMK-3 carbon surface. The basic surface of the carbon thermochemically modified with sodium diclofenac can be the result of the presence of proton acceptors, such as carbon aromatic rings and nitrogen and oxygen surface basic groups [24].

**Figure 2:**
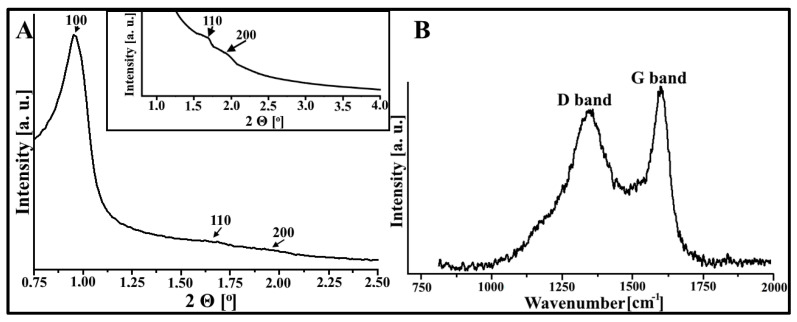
(**A**) The XRD diffractogram of the DF-CMK-3 carbonaceous material. (**B**) Raman bands for the DF-CMK-3 carbon.

Figure 2A presents the XRD diffractogram for the carbonaceous material studied. The diffraction pattern for the DF-CMK-3 carbon consists of three reflections located at various 2Θ angles: 0.95°, 1.69°, and 1.91°. They are related to the following *hkl* lattice planes: 100, 110, and 200, which are characteristic of the ordered hexagonal arrangement (*P6mm*) of mesopores in the CMK-3 porous structure [33]. Moreover, the Raman bands for the carbon studied are displayed in Figure 2B. There are two spectral bands located at 1348 1/cm (D band) and 1600 1/cm (G band). The pattern of the presented Raman spectrum is characteristic of both the CMK-3 and amorphous activated carbons. Also, the intensity ratio of the Raman bands (I_D_:I_G_ = 0.87) indicates that the DF-CMK-3 carbon consists of amorphous carbonaceous rods [34].

The elemental composition of the synthesized carbon obtained by CHN, SEM-EDX, and XPS studies is shown in Table 2. The main element of the studied material is naturally carbon (>90 wt. %). The nitrogen (0.87–1.81 wt. %) and oxygen (4.6–7.4 wt. %) atoms are also present on the surface of the DF-CMK-3 carbonaceous material. The large scatter of the nitrogen and oxygen content on the DF-CMK-3 surface estimated using SEM-EDX and XPS techniques can be attributable to the heterogeneous distribution of heteroatoms in its structure (various heteroatom contents on the carbonaceous surface (up to several nm (XPS)) and in the deeper layers of the studied material (up to several µm (SEM-EDX)) [24].

**Figure 3:**
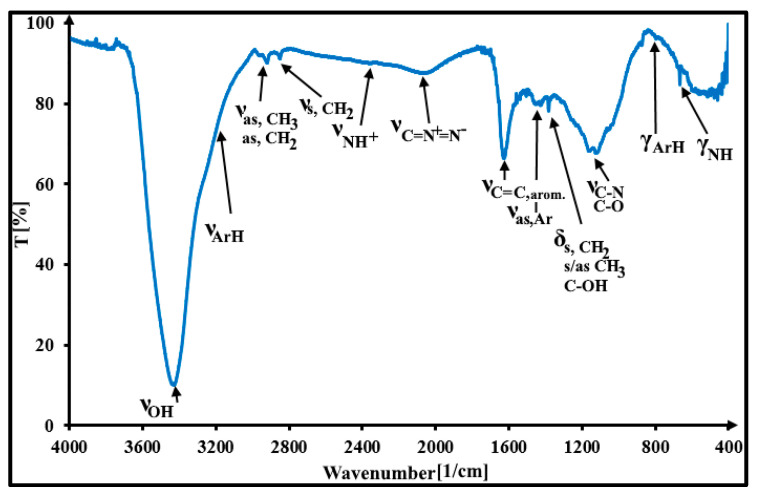
The FT-IR spectrum for the DF-CMK-3 carbon.

**Figure 4:**
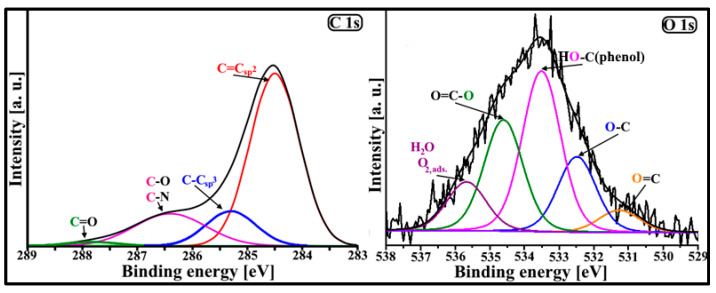
The deconvoluted XPS spectra of C 1s and O 1s core energy levels for the DF-CMK-3 carbon.

It is possible to investigate in detail the kind of surface functional groups present in the DF-CMK-3 carbon through FT-IR and XPS studies. Figure 3 shows the FT-IR spectrum of the studied carbon. There are some spectral bands present, located at 3435 1/cm, 3070-3000 1/cm, 2969 1/cm, 2919 1/cm, 2848 1/cm, 2362 1/cm, 2062 1/cm, 1629 1/cm, 1456 1/cm, 1384 1/cm, 1162-1116 1/cm, 890 1/cm, and 668 1/cm, which correspond to the ν_OH_, ν_ArH_, ν_as,CH3_, ν_as,CH2_, ν_s,CH2_, ν_NH+_, ν_C=(N+)=(N−)_, ν_C=C, arom._, ν_as,Ar_, δ_s/as,CH3;s,CH2_ and δ_C-OH_, ν_C-O,C-N_, γ_ArH_, and γ_NH_, respectively [35,36,37,38]. The FT-IR studies confirmed the presence of basic carbon aromatic rings, aliphatic carbon, and hydroxylamine groups on the surface of the carbon material studied. Figure 4 presents the deconvoluted XPS spectra of C 1s (285.0 eV) and O 1s (534.0 eV) core energy levels for the studied carbon material. Due to the deconvolution of the XPS C 1s core energy level, four signals were obtained, located at 284.5 eV (C=Csp^2^), 285.3 eV (C-Csp^3^), 286.4 eV (C-O/C-N), and 287.8 eV (C=O) [39]. In the case of the O 1s XPS band, however, five signals were obtained, located at 531.0 eV (O=C), 532.4 eV (O-C), 533.5 eV (HO-C(phenol)), 534.7 eV (O=C-O), and 535.6 eV (H_2_O, O_2,ads._) [40,41]. The highest contribution to the C 1s band intensity is related to the C=Csp^2^ groups (67.0%). The contributions of the other two groups (C-Csp^3^ and C-O/C-N) to the C 1s band are 13.5% and 18.1%, respectively. The lowest contribution to the C 1s band intensity is associated with the C=O groups (1.4%). Based on these results, it can be stated that the heteroatoms from sodium diclofenac molecules were successfully introduced into the surface of the carbonaceous material. Contrary to that, the large contribution to the O 1s band intensity is related to the HO-C(phenol) (38.0%) and O=C-O (26.6%) surface groups. Therefore, the chromene and pyron-like oxygen groups, nitrogen groups (e.g., lactam), and aromatic carbon rings can be responsible for the basic character of the surface of the DF-CMK-3 carbon. For comparison, the physicochemical characteristics of the pristine CMK-3 carbon are included in our previous work [30]. 

### 3.2. Characteristics of the GCE/CMK-3/CTAB Sensor

**Figure 5:**
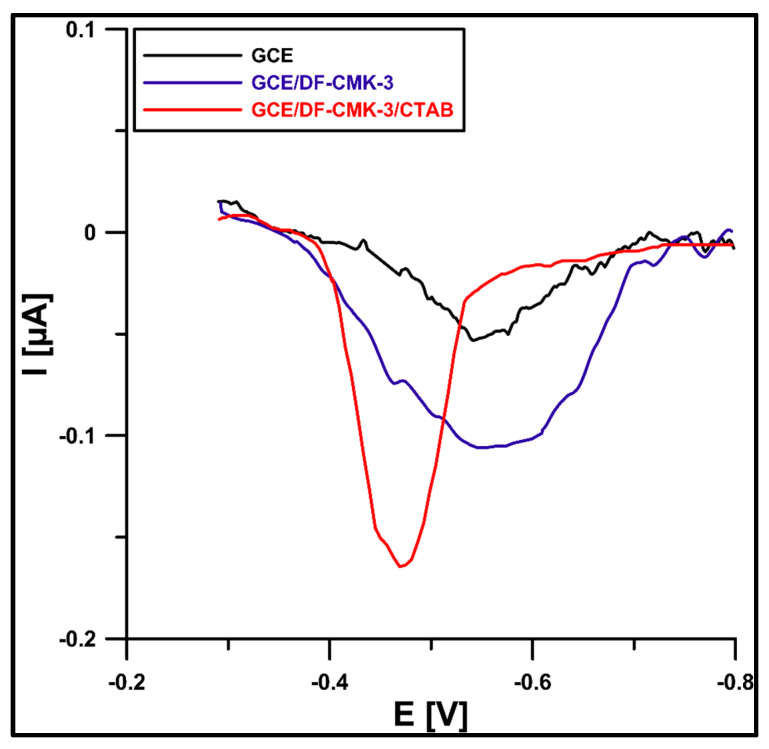
SWAdSV voltammograms of 1 µM ROX at the GCE, GCE/DF-CMK-3, and GCE/DF-CMK-3/CTAB in the solution of 0.075 M acetate buffer (pH of 5.6). Other data: CTAB concentration of 40 mg/L, stirring time of 45 s, f of 100 Hz, E_SW_ of 25 mV, and ΔE of 2 mV.

At the beginning of the research, the SWAdSV technique was applied to describe ROX (1 µM) behavior in 0.075 M acetate buffer (pH of 5.6) at the GCE (glassy carbon electrode), GCE/DF-CMK-3 (GCE modified with the DF-CMK-3 carbon), and GCE/DF-CMK-3/CTAB (GCE modified with the DF-CMK-3 carbon and CTAB). Figure 5 shows that the ROX signal at the GCE/DF-CMK-3/CTAB is 1.6 times higher than at the GCE-DF-CMK-3 and 3.1 times higher than at the GCE. Furthermore, the additional modification of the CTAB contributes to the improvement of the ROX signal shape. The increase in ROX signal due to the application of the DF-CMK-3 layer can be explained by the porosity and surface chemistry of carbon, which contribute to the improvement of the adsorption capacity of the electrode material. Moreover, further modification by the cationic surfactant, cetyltrimethylammonium bromide, resulted in a significant increase in the analytical signal that was observed due to electrostatic interactions between the cationic surfactant and the anion form of ROX in the solution with acidic pH [31].

**Figure 6:**
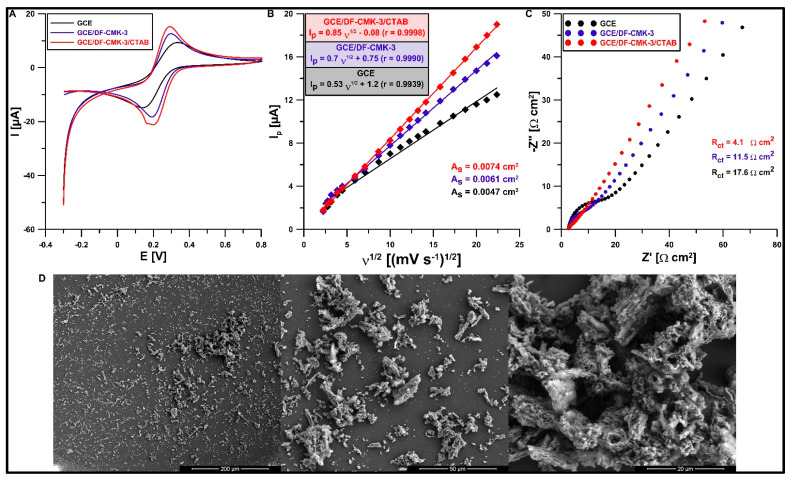
(**A**) CV curves of 5.0 mM K_3_[Fe(CN)_6_] in 0.1 M KCl at the GCE, GCE/DF-CMK-3, and GCE/DF-CMK-3/CTAB (υ of 500 mV/s). (**B**) The relationship between I_p_ and υ^1/2^ at the GCE, GCE/DF-CMK-3, and GCE/DF-CMK-3/CTAB. (**C**) EIS spectra at the GCE, GCE/DF-CMK-3, and GCE/DF-CMK-3/CTAB. (**D**) The SEM images of GCE/DF-CMK-3 for different resolutions.

Another aim was to study the effect of the modification on the electrochemical properties of the electrode using cyclic voltammetry (CV) and electrochemical impedance spectroscopy (EIS). CV curves (ν from 5 to 500 mV s^−1^) and EIS spectra (f from 50,000 to 1 Hz and at a potential of 0.2 V) were registered in KCl solution (0.1 M) with the addition of K_3_[Fe(CN)_6_] (5 mM). Figure 6A shows the CV curves recorded at the GCE, GCE/DF-CMK-3, and GCE/DF-CMK-3/CTAB for ν of 500 mV s^−1^. Improvement of the Fe(II) oxidation signal and the relative separation of the oxidation and reduction peaks (χ^0^) were observed with the modification of the GCE surface. The χ^0^ values were 2.98, 1.74, and 1.41 for the GCE, GCE/DF-CMK-3, and GCE/DF-CMK-3/CTAB, respectively, which indicates better electron transfer kinetics for the GCE/DF-CMK-3/CTAB, where χ^0^ is closer to the theoretical value (χ^0^ = 1). Based on the obtained CVs, the relationship between the anode peak current (I_p_) and the square root of ν was studied (Figure 6B). On the basis of these results, the active area (A_s_) of both electrodes was calculated using the Randles–Sevcik equation [42]. The obtained results showed an increase in the A_s_ of the GCE/DF-CMK-3/CTAB compared to the GCE/DF-CMK-3 and GCE (0.074 vs. 0.061 cm^2^ and 0.074 vs. 0.047 cm^2^, respectively). Figure 6C shows the EIS spectra where the GCE/DF-CMK-3/CTAB has a lower charge transfer resistance (R_ct_) than the GCE/DF-CMK-3 and GCE (4.1 vs. 11.5 Ω cm^2^ and 4.1 vs. 17.6 Ω cm^2^, respectively). Figure 6D displays the SEM images of the GCE/DF-CMK-3 surface morphology. As can be seen, the GCE surface is covered with carbon particles of various sizes. Moreover, the worm-like morphology of the particles is partially deformed, which can be the result of the thermochemical modification with sodium diclofenac.

### 3.3. Optimization of the GCE/DF-CMK-3/CTAB Fabrication Procedure

**Figure 7:**
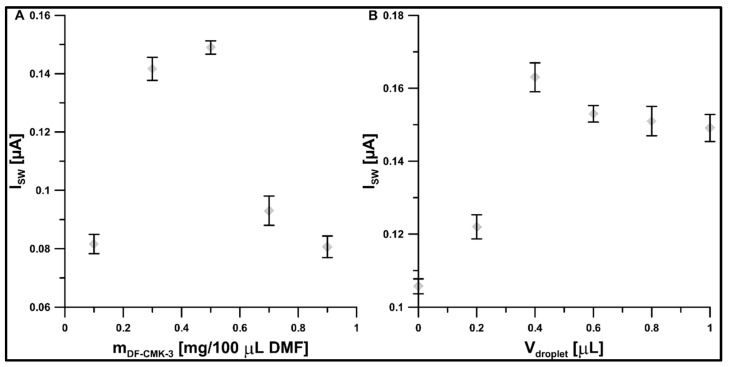
The impact of (**A**) amounts of DF-CMK-3 in the suspension, and (**B**) drop volume on the 1 µM ROX. Other parameters are as in Figure 6.

The effect of different contents of DF-CMK-3 in the carbon suspension (the weight of DF-CMK-3 in 100 µL of DMF) on the 1 µM ROX signal was evaluated. The electrode surface was additionally modified with CTAB, whose concentration of 40 mg/L had been optimized in our previous work [20]. The results are shown in Figure 7A. The ROX signal increased with increasing content of DF-CMK-3 up to 0.5 mg/100 µL DMF and then decreased. The signal increase is most likely related to the improvement of the adsorption capacity of the surface with respect to ROX, while the decrease is attributable to the blocking of the surface by too thick layers of the carbon material. A mass of 0.5 mg was selected for further testing.

Subsequently, the impact of the drop volume (0–1 µL) on the 1 µM ROX signal was tested. The results demonstrate that the DF-CMK-3 layer fabricated by 0.2 µL suspension of 0.5 mg DF-CMK-3 in 100 µL DMF was too thin for ROX analysis (Figure 7B). The highest ROX peaks were obtained for a drop volume of 0.4 µL and it was chosen for further experiments.

### 3.4. Selection of Electrolyte and Study of ROX Behavior in It

**Figure 8:**
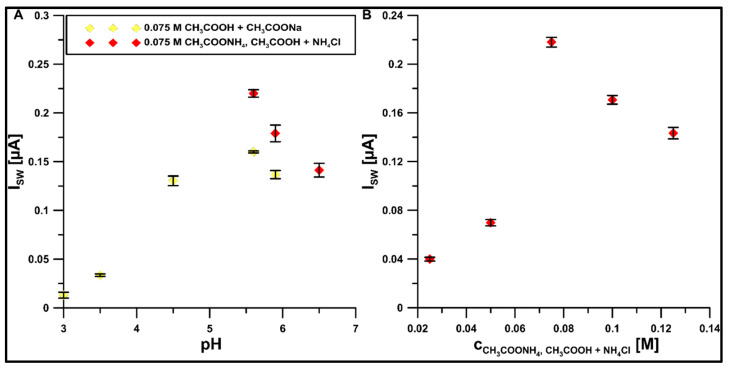
The impact of (**A**) type and pH value, and (**B**) concentration of the electrolyte on the 1 µM ROX. Other parameters are as in Figure 6.

To further improve the electrochemical properties of the GCE/DF-CMK-3/CTAB for ROX analysis, the composition of the supporting electrolyte (CH_3_COONH_4_, CH_3_COOH, and NH_4_Cl) was proposed [30]. As shown in Figure 8A, the improvement of the conductivity of the supporting electrolyte by changing the typical acetate buffer (CH_3_COONa and CH_3_COOH) to buffer solution (CH_3_COONH_4_, CH_3_COOH, and NH_4_Cl) amplifies the ROX signal to 175% of its original value. Moreover, the studies showed, based on the ROX peak current, that the best pH value of the supporting electrolyte is 5.6 (Figure 8A), and the best concentration is 0.075 M (Figure 8B).

**Figure 9:**
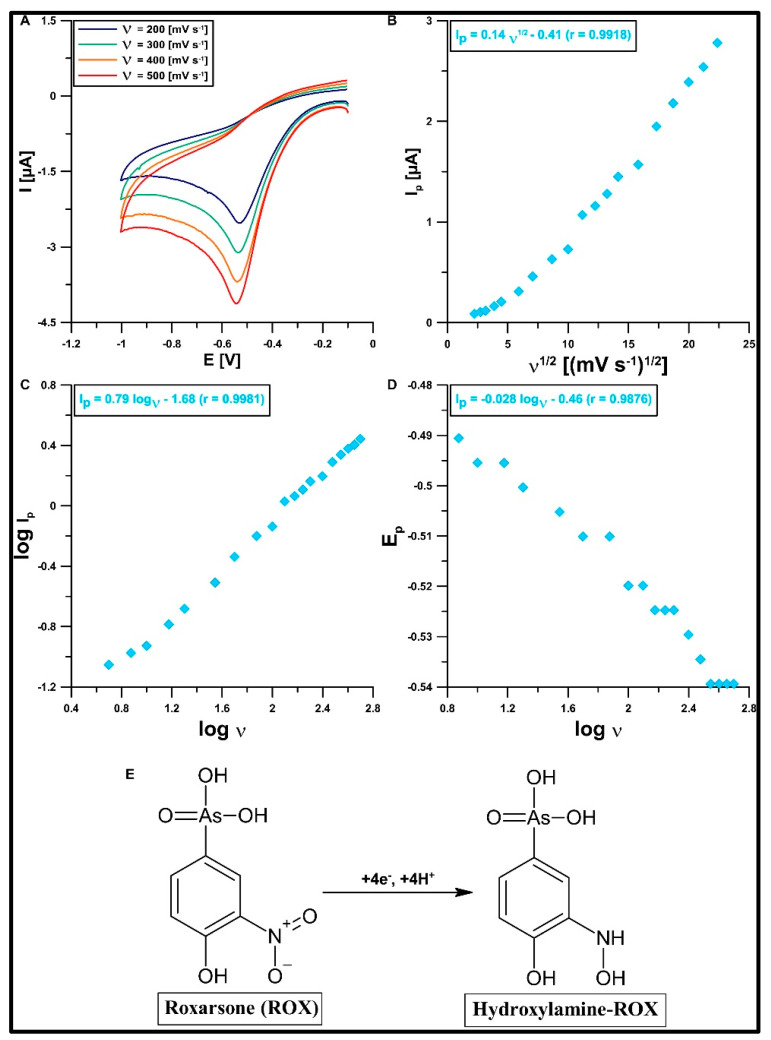
(**A**) CVs obtained at the GCE/DF-CMK-3/CTAB in 0.1 M buffer solution (CH_3_COONH_4_, CH_3_COOH, and NH_4_Cl) of pH = 5.6, 40 mg/L CTAB, and 50 µM ROX. The dependence between I_p_ and ν^1/2^ (ν from 5 to 500 mV s^−1^) (**B**), log I_p_ and log ν (**C**), E_p_ and log ν (**D**), and ROX reduction mechanism (**E**).

The selected conditions (type, pH, and concentration of the electrolyte) were used for the CV studies. In total, 50 µM of ROX was added to the electrolyte and CVs were recorded, varying ν from 5 to 500 mV s^−1^. The CV curves obtained at the ν values of 200, 300, 400, and 500 mV s^−1^ are shown in Figure 9A. One reduction peak, which moves as the scanning speed increases, is visible in the CV curves. It can be seen that we are dealing with an irreversible reduction process of ROX. The nature of this process at the GCE/DF-CMK-3/CTAB is determined by the dependencies between I_p_ and ν^1/2^ (Figure 9B) as well as log I_p_ and log ν (Figure 9C). The first dependency is close to linear, with the slope of the curve being between 0.5 and 1. These results confirm that the nature of the electrode process is not purely adsorptive, but it is a mixed adsorption–diffusion-controlled process. The ROX reduction mechanism is described in the literature [20]. This process at the GCE/DF-CMK-3/CTAB involves four electrons (which is confirmed based on the relationship between the reduction peak potential of ROX (Ep) and log υ; Figure 9D) and four protons, and it is associated with the reduction of the nitro group to hydroxylamine (Figure 9E).

### 3.5. Optimization of Procedure Parameters

As described above, the nature of the electrode process is not purely adsorptive, but it is a mixed adsorption–diffusion-controlled process. Therefore, the influence of the accumulation potential in the range from 0.5 to −0.5 on the 1 µM ROX signal was investigated in the first step of the experiments. The applied potential was not observed to affect the signal and hence, further measurements were carried out in an open circuit. However, changing the accumulation time to 90 s did increase the signal. Moreover, the lack of mixing at the accumulation stage (90 s) contributed to the improvement of the signal shape and its increase by an additional 20%. This is most likely due to the fact that the lack of mixing facilitates the formation of a surfactant layer on the electrode surface and the electrostatic attraction of ROX in the anionic state.

During the next stage of this research, the effect of the parameters of the signal recording technique (SWV) on the intensity of the 1 µM ROX signal was examined. In the first stage, the frequency (f) was changed in the range from 25 to 250 Hz with constant values of the amplitude (E_SW_) of 25 mV and the step potential (ΔE) of 2 mV. The ROX signal was found to reach the highest value at the f of 200 Hz. Next, the E_SW_ was changed in the range from 20 to 120 mV, with an optimized f of 200 Hz and a constant ΔE of 2 mV. The E_SW_ of 75 mV was selected for further testing based on the intensity of the ROX signal. In the last stage, the ΔE was optimized and changes in its value were made in the range from 2 to 18 mV. The ROX signal was found to increase to a value of 6 mV and then sharply decrease.

### 3.6. Selectivity and Analytical Characteristics

**Figure 10:**
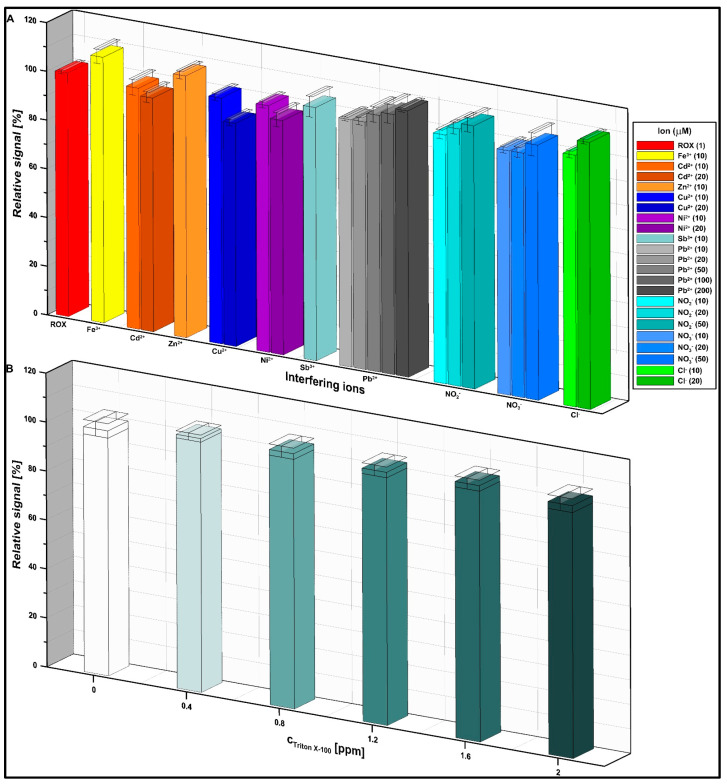
The effect of selected ions (**A**) and Triton X-100 (**B**) on the 1 µM ROX signal. The SD was calculated for n = 3.

The selectivity of the measurement procedure was checked during the conducted experiments. The influence of selected ions (Fe^3+^, Cd^2+^, Zn^2+^, Cu^2+^, Ni^2+^, Sb^3+^, Pb^2+^, NO_2_^−^, NO_3_^−^, and Cl^−^) and the model surfactant, Triton X-100, on the 1 µM ROX peak current was investigated. The results are shown in Figure 10. The excess of the tested ions, which do not change the signal by 10%, are given in brackets. Likewise, changes in the current intensity of the ROX peak greater than ±10% were not observed in the presence of 2 ppm of Triton X-100.

**Figure 11:**
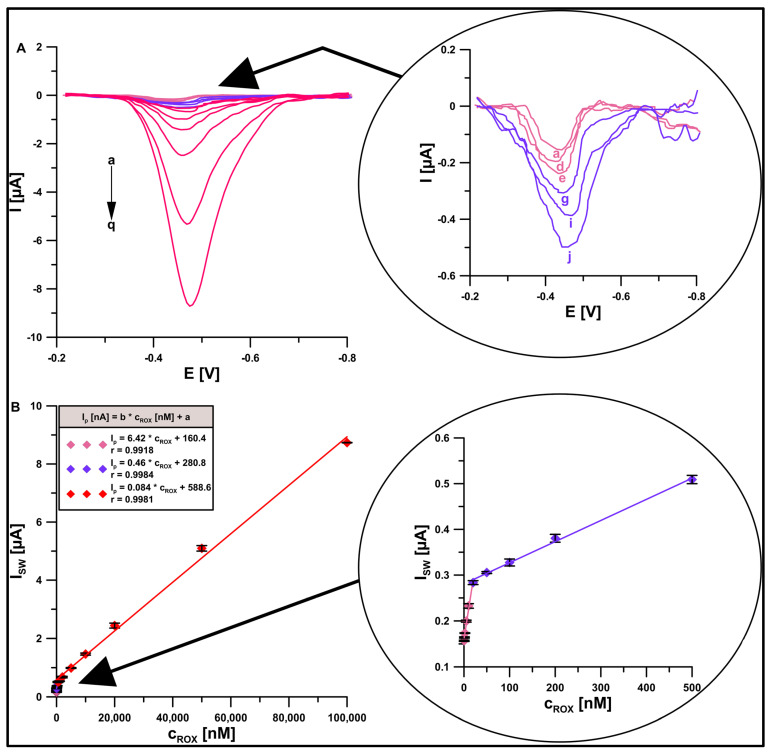
(**A**) SWAdSV curves at the GCE/DF-CMK-3/CTAB in 0.1 M buffer solution (CH_3_COONH_4_, CH_3_COOH, and NH_4_Cl) of pH = 5.6, 40 mg/L CTAB, and ROX (a→q, 0.0005, 0.001, 0.002, 0.005, 0.01, 0.02, 0.05, 0.1, 0.2, 0.5, 1, 2, 5, 10, 20, 50, 100 µM). (**B**) Linear calibration plots of ROX. The procedure parameters: t = 90 s, f = 200 Hz, E_SW_ = 75 mV, and ΔE = 6 mV. The SD was calculated for n = 3.

In the next part of this study, measurements were made with respect to the ROX calibration curve. The SWAdSV signals were recorded for increasing ROX concentrations from 0.5 to 100,000 nM. The calibration curve was observed to have three linear ranges (0.5–20 nM, 20–500 nM, and 500–100,000 nM) (Figure 11). The limits of detection (LOD) and quantification (LOQ) calculated as LOD = 3SD_a_/b and LOQ = 10SD_a_/b [43] were 9.6 × 10^−11^ and 3.2 × 10^−10^ M, respectively. Table 3 compares the analytical parameters of the developed procedure with those previously described in the literature for other voltammetric sensors [10,11,12,13,14,15,16,17,18,19,20,21,22,23]. As can be seen, we obtained a very wide range of linearity of the calibration curve and additionally the lowest limit of detection in comparison to those that had been described in the literature thus far. It should be emphasized that in this work, the analytical parameters at the GCE/DF-CMK-3/CTAB were significantly improved compared to our work on the GCE/CTA [20]. The relative standard deviation for the signals for all points on the calibration curve ranges from 0.08 to 4.05%, which indicates very good repeatability of the analytical signal.

### 3.7. Practical Application

The practical application of the developed procedure was checked by analyzing enriched samples of river water and municipal wastewater. The samples were diluted 10-fold and analyzed using the method of standard addition. It should be noted that sample dilution is an easy way to minimize interference from the matrix, but it can only be used if the analytical procedure has a low limit of detection and quantification. The recoveries from 96 to 102% prove the high accuracy of the proposed procedure (Table 4). Furthermore, the values of the coefficient of variation (3.31–3.92) indicate good repeatability of the ROX signal.

## 4. Conclusions

In summary, this study proposed a selective and sensitive SWAdSV procedure using a glassy carbon sensor modified with diclofenac-impregnated mesoporous carbon and a cationic surfactant, CTAB (GCE/DF-CMK-3/CTAB), for trace analysis of the arsenic drug roxarsone (ROX). The EIS and CV results showed numerous advantages of subjecting the SPCE to the modification process, including a significant increase in the active surface of the working electrode, a decrease in the resistance of charge transfer, as well as improvement in electron transfer kinetics and ROX reduction peak. In addition, the SEM images show that the electrode is covered with carbon particles of various sizes. Moreover, the worm-like morphology of the particles is partially deformed, which can be the result of the thermochemical modification with sodium diclofenac. The developed procedure is characterized by excellent sensitivity and allows a very low limit of detection (9.6 × 10^−11^ M) with a wide range of linearity, from 5.0 × 10^−10^ to 1.0 × 10^−4^ M, to be achieved. The usefulness of the proposed procedure was confirmed by successfully determining ROX in spiked samples of treated municipal wastewater and river water.

## Figures and Tables

**Table 1:** The specific surface area (S_BET_), total pore volume (V_T_), pore diameter (d_BJH_), Raman intensities ratio, zeta potential, and pH of 1 mM KCl for the DF-CMK-3 carbon.

S_BET_ (m^2^/g)	V_T_ (cm^3^/g)	d_BJH_ (nm)	I_D_:I_G_ (au)	ζ (mV)	pH (au)
690 ± 11 *	0.79 ± 0.01 *	3.4 ± 0.1 *	0.87 ± 0.01 *	12.3 ± 0.1 *	7.77 ± 0.15 *

* SD from 3 replicates.

**Table 2:** The elemental composition of the DF-CMK-3 carbon obtained using CHN, SEM-EDX, and XPS studies.

CHN			SEM-EDX ^#^			XPS ^$^	
C (wt. %)	H (wt. %)	N (wt. %)	C (wt. %)	O (wt. %)	N (wt. %)	C (wt. %)	O (wt. %)
93.5 ± 2.7 *	0.63 ± 0.01 *	0.87 ± 0.02 *	92.9 ± 0.5 *	4.6 ± 0.3 *	1.81 ± 0.04 *	91.3 ± 1.7 *	7.4 ± 0.9 *

* SD from 3 replicates, ^#^ Na, Cl, Si, S < 1 wt. %, ^$^—Si < 1.5 wt. %.

**Table 3:** Voltammetric procedures for ROX determination.

Technique (Sensor)	Linear Range (µmol L^−1^)	LOD (µmol L^−1^)	Matrix Type	Ref.
DPV (WS_2_NSs/SPCE)	0.05–489.3	0.030	meat	[10]
DPV (WS_2_ NRs/N-rGOs/SPCE)	0.1–442.6	0.075	human serum, urine, and pharmaceutical sample	[11]
DPV (ZnSnO_3_@GO/GCE)	0.01–453.4	0.0043	chicken and soil samples	[12]
DPAdSV (MCPME)	3.8–190.0	0.19	poultry feed and poultry litter	[13]
DPAdSV (CMCPE)	0.1–1.0	0.10	poultry drinks and veterinary products (tablets)	[14]
SWV (Au/ErGO/SPCE)	1.0–1000.0	0.014	meat	[15]
DPV (2D-AC/GCE)	0.76–474.0	0.0015	blood serum	[16]
DPV (Tm-BTC MOF/GCE)	0.00015–770.0	0.0001	food samples	[17]
DPV (CoMn_2_O_4_-500)	0.01–0.84 and 0.84–1130.0	0.002	river water	[18]
DPV (LaMoO)	0.025–2650.0	0.012	food analysis	[19]
SWAdSV (GCE/CTAB)	0.001–0.02 and 0.02–20.0	0.00013	river water	[20]
CV and DPV (DyVO_4_/SPCE)	0.01–21.0 and 36.0–264.0	0.0009	water, urine, chicken, and egg	[21]
DPV (CrNiCo-P/GCN/GCE)	1.0–413.0	0.031	chicken and swine meat	[22]
DPV (MoS_2_/S-Ti_3_C_2_/LGE)	0.01–875.01	0.0023	asarum sieboldii, urine, and blood serum	[23]
SWAdSV (GCE/DF-CMK-3/CTAB)	0.0005–0.02, 0.02–0.5, and 0.5–100.0	0.000096	river water and municipal wastewater	This work

Techniques: DPV—differential pulse voltammetry; DPAdSV—differential–pulse adsorptive stripping voltammetry; SWV—square-wave voltammetry; CV—cyclic voltammetry; SWAdSV—square-wave adsorptive stripping voltammetry. Sensor: WS_2_NSs/SPCE—screen-printed carbon electrode modified with tungsten disulfide nanosheets; WS_2_ NRs/N-rGOs/SPCE—screen-printed carbon electrode modified with tungsten disulfide nanorods-decorated nitrogen-doped reduced graphene oxide-based nanocomposite; ZnSnO_3_@GO/GCE—glassy carbon electrode modified with perovskite-structured ZnSnO_3_@GO nanocomposites; MCPME—modified carbon paste microelectrode; CMCPE—chemically modified carbon paste electrode; Au/ErGO/SPCE—screen-printed carbon electrode modified with gold-coated electrochemically reduced graphene oxide; 2D-AC/GCE—glassy carbon electrode modified with two-dimensional activated carbon; Tm-BTC MOF/GCE—glassy carbon electrode modified with thulium (III)-benzene 1,3,5-tricarboxylic acid-based metal-organic framework; CoMn_2_O_4_-500—screen-printed carbon electrode modified with three-dimensional cobalt manganate with flowerlike structures; LaMoO—screen-printed carbon electrode modified with roselike lanthanum molybdate electrocatalyst; GCE/CTAB—glassy carbon electrode modified with cetyltrimethylammonium bromide; DyVO_4_/SPCE—screen-printed carbon electrode modified with dysprosium vanadate; CrNiCo-P/GCN/GCE—glassy carbon electrode modified with graphitic carbon nitride nanosheets coated with multi-metallic phosphides; MoS_2_/S-Ti_3_C_2_/LGE—laser-induced graphene electrode modified with MoS_2_ sphere-combined sulfur-doped Ti_3_C_2_ MXene nanocatalyst; (GCE/DF-CMK-3/CTAB)—a glassy carbon electrode modified with diclofenac-impregnated mesoporous carbon and cetyltrimethylammonium bromide.

**Table 4:** The ROX analysis in real samples.

Sample	Added (μmol L^−1^)	Determined ± SD (n = 3) (μmol L^−1^)	Coefficient of Variation (%)	Recovery (%)
Vistula River	0.02 0.2	0.0204 ± 0.000743 0.192 ± 0.00753	3.64 3.92	102.0 96.0
Purified municipal wastewater	0.02 0.2	0.0199 ± 0.000666 0.204 ± 0.00675	3.35 3.31	99.5 102.0

Coefficient of variation [%] = (SD × 100)/Determined, Recovery [%] = (Determined × 100)/Added.

## Data Availability

Not applicable.
